# Morphologically normalized left ventricular motion indicators from MRI feature tracking characterize myocardial infarction

**DOI:** 10.1038/s41598-017-12539-5

**Published:** 2017-09-25

**Authors:** Paolo Piras, Luciano Teresi, Paolo Emilio Puddu, Concetta Torromeo, Alistair A. Young, Avan Suinesiaputra, Pau Medrano-Gracia

**Affiliations:** 1 Dipartimento di Scienze Cardiovascolari, Respiratorie, Nefrologiche, Anestesiologiche e Geriatriche, Sapienza Università di Roma, Via del Policlinico 155, 00186 Roma, Italy; 2 Dipartimento di Ingegneria Strutturale e Geotecnica, Sapienza Università di Roma, Via Eudossiana 18, 00184 Roma, Italy; 3 Dipartimento di Matematica e Fisica, LaMS–Modeling & Simulation Lab, Via della Vasca Navale 84, Università Roma Tre, Roma, 00146 Roma, Italy; 4 Department of Anatomy and Medical Imaging, School of Medical Sciences, Faculty of Medical and Health Sciences, University of Auckland, 85 Park Rd, Grafton, Auckland, 1023 New Zealand

## Abstract

We characterized motion attributes arising from LV spatio-temporal analysis of motion distributions in myocardial infarction. Time-varying 3D finite element shape models were obtained in 300 Controls and 300 patients with myocardial infarction. Inter-individual left ventricular shape differences were eliminated using parallel transport to the grand mean of all cases. The first three principal component (PC) scores were used to characterize trajectory attributes. Scores were tested with ANOVA/MANOVA using patient disease status (Infarcts *vs*. Controls) as a factor. Infarcted patients had significantly different magnitude, orientation and shape of left ventricular trajectories in comparison to Controls. Significant differences were found for the angle between PC scores 1 and 2 in the endocardium, and PC scores 1 and 3 in the epicardium. The largest differences were found in the magnitude of endocardial motion. Endocardial PC scores in shape space showed the highest classification power using support vector machine, with higher total accuracy in comparison to previous methods. Shape space performed better than size-and-shape space for both epicardial and endocardial features. In conclusion, LV spatio-temporal motion attributes accurately characterize the presence of infarction. This approach is easily generalizable to different pathologies, enabling more precise study of the pathophysiological consequences of a wide spectrum of cardiac diseases.

## Introduction

Statistical shape modeling is a powerful tool for visualizing and quantifying geometric and functional patterns of variation in the heart. Specific pathological conditions directly affect 3D and 4D (i.e. 3D + time) shape characteristics. Characterization of these effects is essential to understand morphological and dynamic differences between healthy and diseased samples. In recent years, statistical shape analysis has been applied to a variety of diseases such as aortic regurgitation^1^, hypertrophic cardiomyopathy^2–4^ and myocardial infarction (MI)^5–7^. It has been estimated that in 2004, 12.2% of worldwide deaths were from ischemic heart disease^8^, and this is the leading cause of death in high- or middle-income countries and second only to lower respiratory infections in lower- income countries.

After myocardial infarction, the heart remodels in response to physiological challenges. The degree and type of remodeling provides important diagnostic information for therapeutic management. Recently, the extent of this remodeling was explored using finite element modeling^4^ (FEM); for example, after an acute MI, the left ventricle (LV) becomes more spherical^9^. In particular, probabilistic models of LV^6,7^ showed that statistical shape analysis can predict a patient’s disease (i.e. MI) status. In a recent computational challenge, eleven strategies were proposed to characterize MI from morphological variation using heart models^7,10^. However, none of these studies exploited the full temporal dynamics of LV motion, as only two time points were provided (end-diastole and end-systole). Dynamic analysis involves the characterization of the entire LV cycle, thus capturing spatio-temporal characteristics. Using individual shapes occurring at comparable times in the context of one heart beat furnishes an invaluable data structure for studying pathophysiological consequences of a variety of diseased conditions. This allows analysis of the motion patterns and detecting their attributes, i.e. discovering the features of the morphological trajectory. This trajectory analysis is relatively new in cardiology^1–3^ and arises from the translation of the concept of homology from the anatomical to the temporal domain.

Recently, 3D Speckle Tracking Echocardiography (3DSTE)^11^ and cine magnetic resonance (MR) have become widely used in studies aimed at modelling the three-dimensional shape of human heart. 3DSTE is more reliable in terms of temporal resolution, whereas MR has greater geometric accuracy^12^. In the future, fusion of 3DSTE and MR could enable better understanding of cardiac mechanics^13^.

The main aim of this study was to examine differences in motion trajectories between asymptomatic controls and MI cases. In contrast to previous studies^5,6^, where only diastolic and systolic states were evaluated, the entire set of shapes (30 normalized time-points) was exploited, comprising the full LV heartbeat.

A trajectory analysis was performed which combines geometric morphometrics and dimension reduction techniques to enable a concise quantitative description of 3D motion patterns from MR feature tracking studies.

## Results

### Linear Shift + PCA

**Figure 1:**
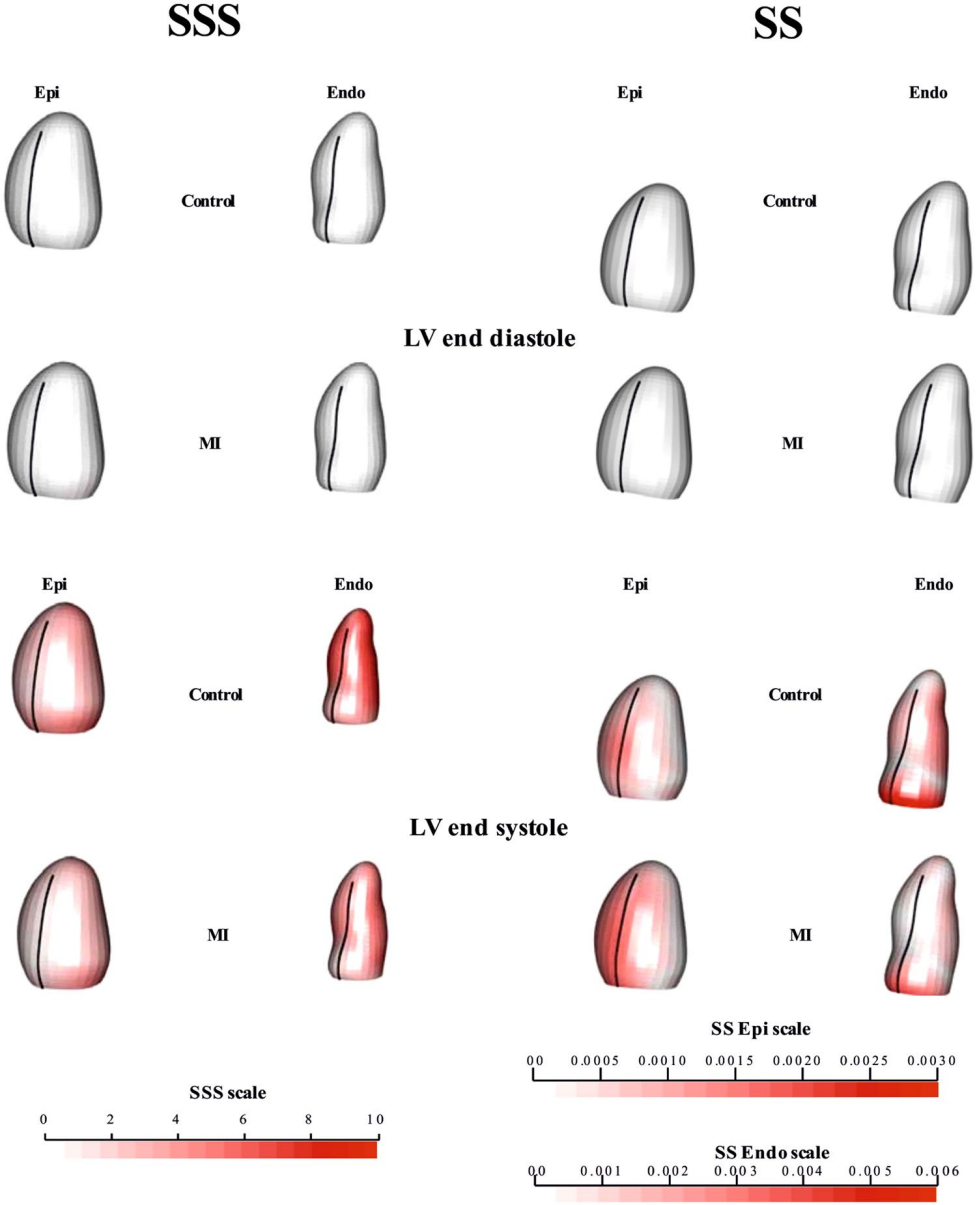
Mean shapes occurring at end-diastole and end-systole after Parallel Transport (PT). Colors in end- systole refer to the difference from end-diastolic state (that is white = undeformed, red = deformed). The colormap ranges from white (=no deformation) to red (max) and refers to || *xM*-*x* || with *xM* the position at end-diastole and x its position at end-systole. As in SS the shapes are scaled to unit size and the deformations of epicardium and endocardium have different magnitudes, different color scales are used. The black line indicates the position of the inter-ventricular septum. See Supplementary Files 1 and 2 for dynamic visualizations.

**Figure 2:**
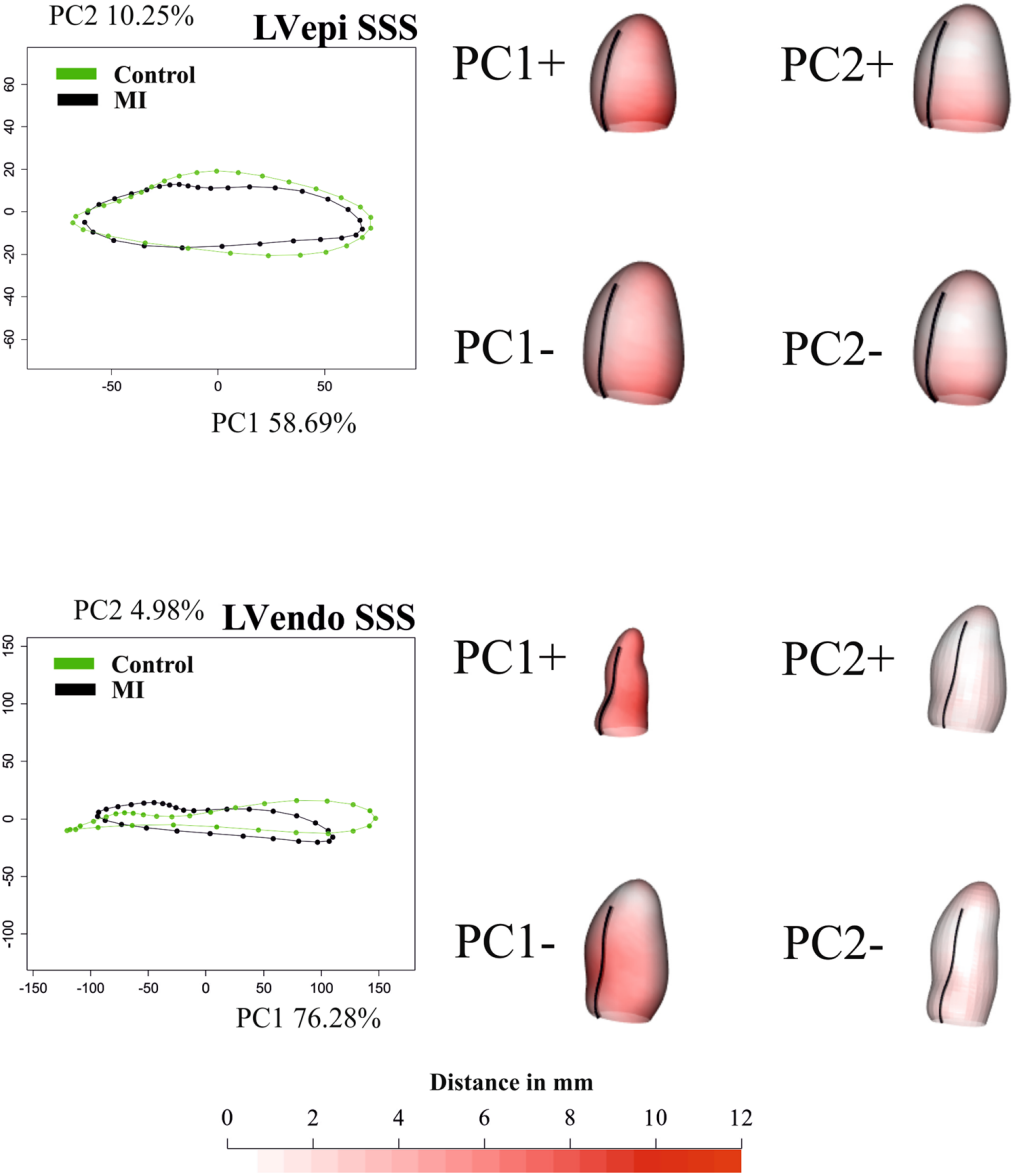
Results of GPA + PT + PCA for epicardium and endocardium in SSS. For visualization purposes, only one per-group mean trajectory (300 Controls vs 300 MI) is shown. The colormap ranges from white (=no deformation) to red (=max) and refers to || *xM*-*x* || with *xM* the position at the Grand Mean and x its position at the PC axis extreme. The black line indicates the position of inter-ventricular septum. The first three PCs are used as “landmarks” in the trajectory analysis taking into account all 600 trajectories present in the dataset. See Supplementary Files 3 and 4 for dynamic visualization of trajectories and associated shape changes. Shapes are predicted at the positive and negative axes’ extremes.

**Figure 3:**
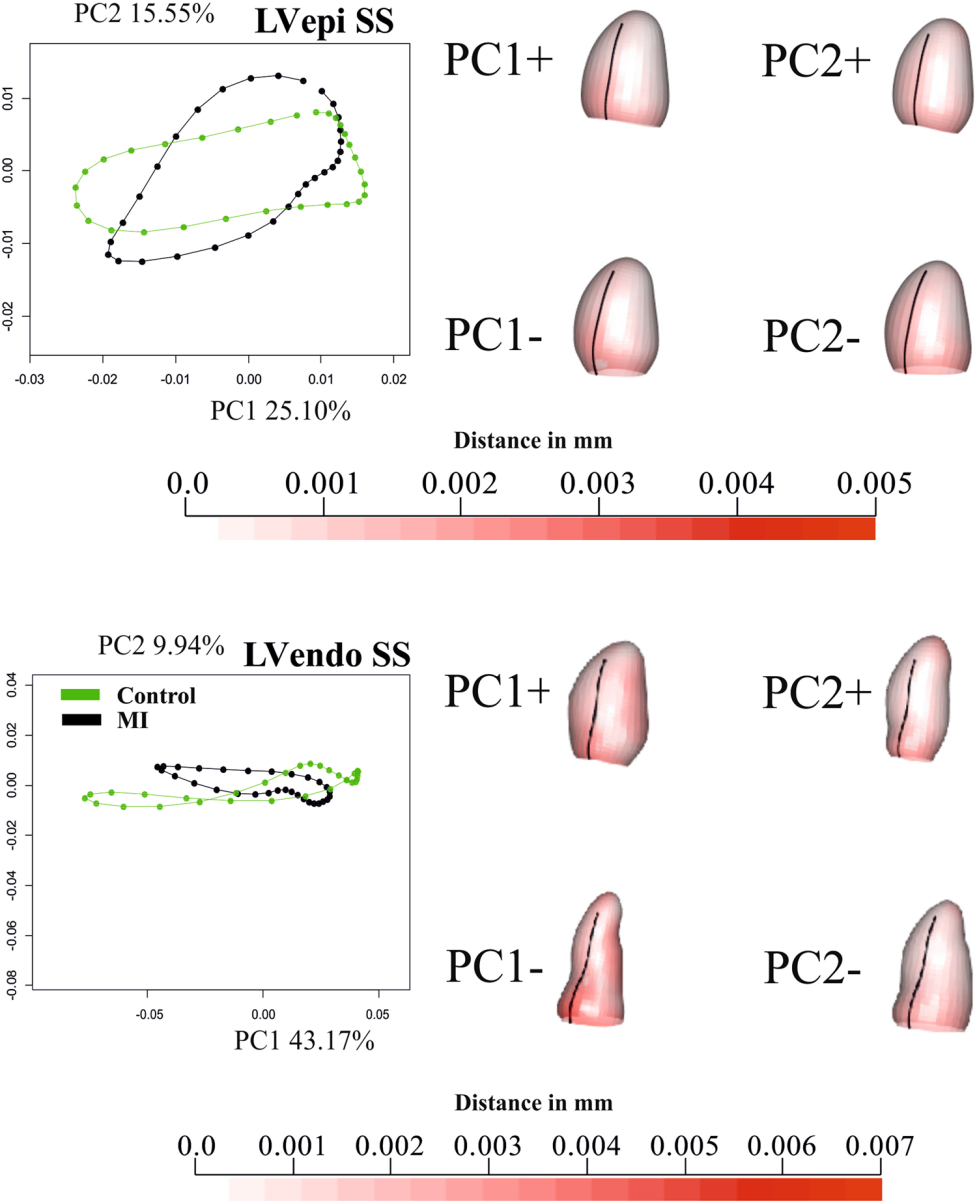
Results of GPA + PT + PCA for epicardium and endocardium in SS. For visualization purposes, only one per-group mean trajectory (300 Controls vs 300 MI) is shown. The colormap ranges from white ( = no deformation) to red (max) and refers to || *xM*-*x* || with *xM* the position at the Grand Mean and x its position at the PC axis extreme. As in SS the shapes are scaled to unit size and the deformations of epicardium and endocardium have different magnitudes, different color scales are used. The black line indicate the position of inter-ventricular septum. The PC scores of first three PCs are used as “landmarks” in the trajectory analysis taking in to account all 600 trajectories present in the dataset. See Supplementary Files 5 and 6 for dynamic visualization of trajectories and associated shape changes. Shapes are predicted at actual positive and negative axes extremes.

Figure 1 shows the deformations of Control and MI starting from ED (for visualization ED is treated as un-deformed) for transported data in both SSS and SS. Supplementary Files 1 and 2 show the corresponding animations. In SSS, Controls showed a larger deformation and contraction relative to MI. This was more evident on the endocardial surface than on the epicardium. SS analysis also showed that the endocardium deforms more than the epicardium, experiencing a more marked affine transformation. In fact, the endocardium in SS appears more elongated in systole compared to the epicardium. Figures 2 and 3 illustrate, separately for the endocardium and epicardium, the global mean trajectories in the space identified by the first two PC scores. In SSS, the variances of PC1 (endocardium and epicardium) scores were much larger than that PC2 or PC3, since they contain size (endocardium: PC1, 76.28%; PC2, 4.98%; PC3, 2.58%; epicardium: PC1, 58.69%; PC2, 10.25%; PC3, 4.01%). In the case of SS, instead, variances were less concentrated due to the fact that size is no longer a direct influencing factor in ordination space (endocardium: PC1, 43.1%; PC2, 9.94%; PC3, 6.18%; epicardium: PC1, 25.10%; PC2, 15.56%; PC3, 7.77%). Again, stronger differences were detectable in the endocardium, which more visibly separated Controls from MI for shape, size and direction of trajectories. Deformations associated with PC axes are also displayed. Supplementary Files 3 and 4 show these trajectories in the space of the first three PC scores, where differences were more evident for endocardial trajectories between Controls and MI. Supplementary Files 5 and 6 show, dynamically, the deformations associated with the first three PC scores. For clarity of interpretation, due to the small amount of variance explained by PC2 and PC3 in SSS, the corresponding animation deformations of these axes are magnified, respectively 2 and 3 times. The elliptical trajectories were oriented very differently between the two groups and the extent of variation of the MI trajectory is higher than in Controls. One expected consequence of doing analyses in both SSS and SS is that PC1 of SSS was represented mainly by “size change”; therefore the other PCs are, approximately, scale free. In fact, PC1 in SS was very similar, in terms of morphological expression, to PC2 in SSS. The other PCs in both SS and SSS analyses, however, were not correlated.

### Trajectory Attributes

**Figure 4:**
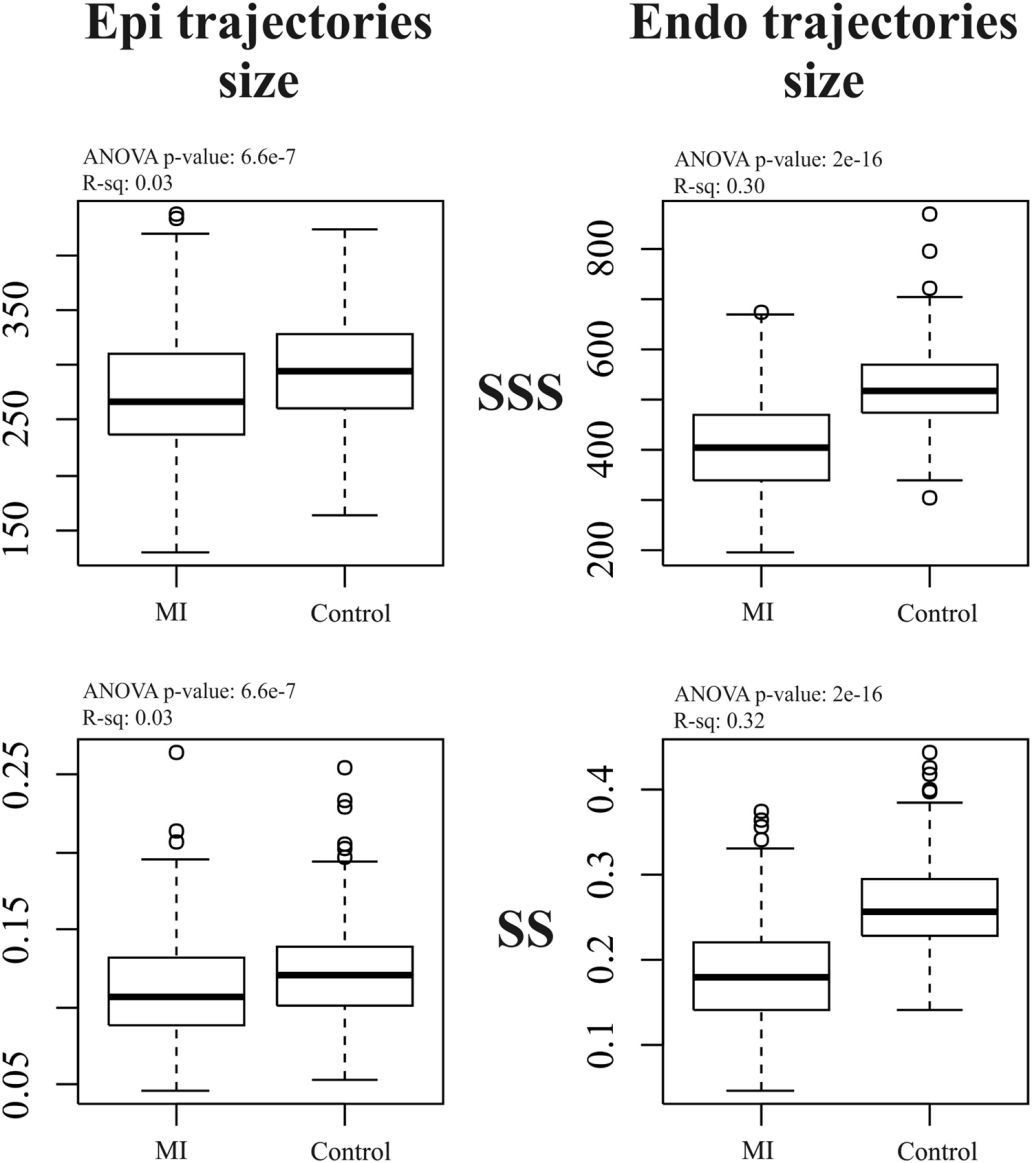
The difference in trajectories size among groups.

As stated above, we contrast the shape, size and orientation attributes of trajectories. Figure 4 shows differences among Controls and MI for endocardial and epicardial trajectory size in SSS and SS. ANOVAs were all significant but the R-squared was larger for endocardial trajectory, indicating a larger effect size as also indicated by the corresponding boxplot. In addition, for the endocardium, SS performed better than SSS.

**Figure 5:**
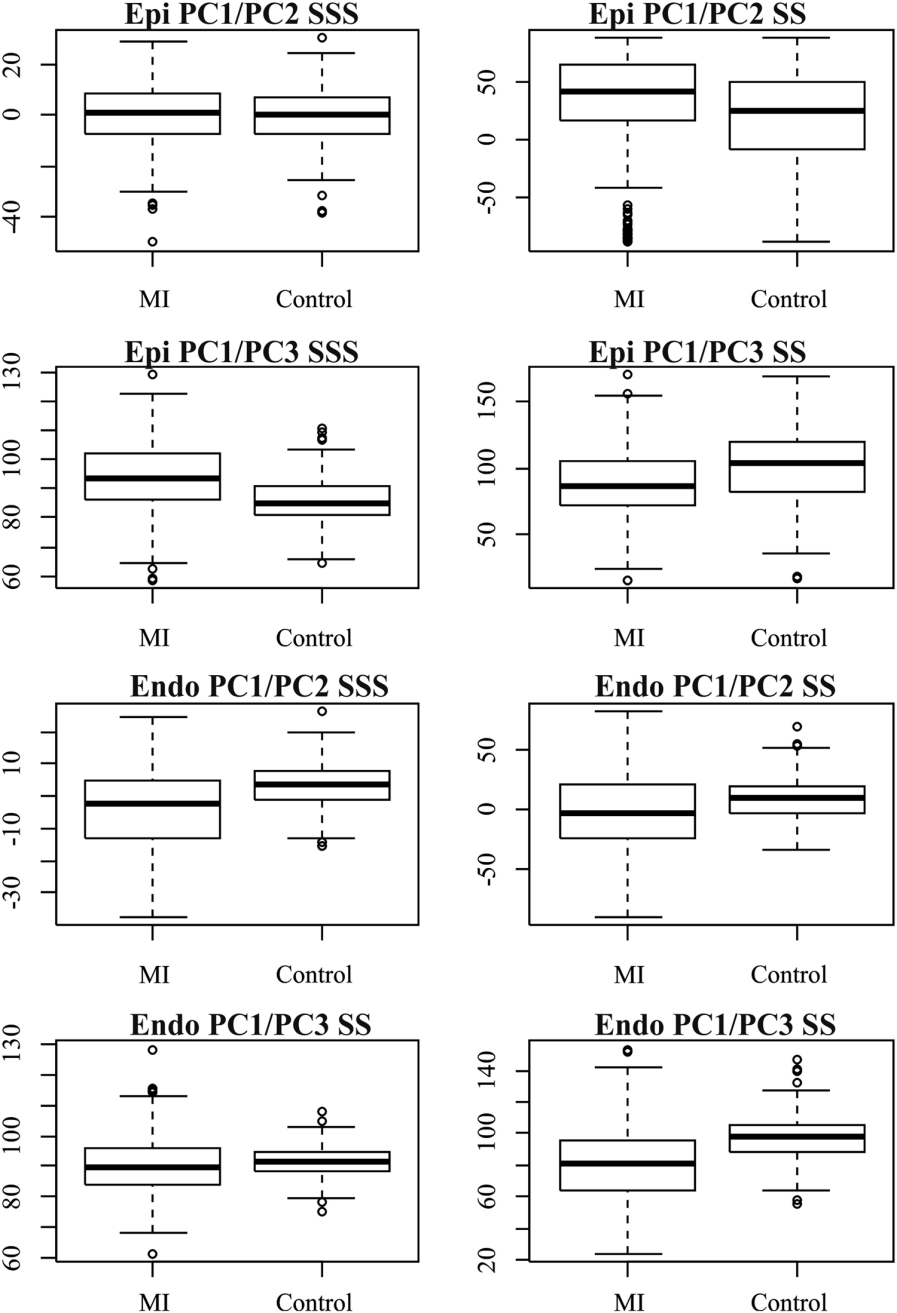
Angle distributions in SSS and SS.

Figure 5 shows the results relative to angle differences. Table 1 shows corresponding ANOVAs. Although most of them were significant, larger effect sizes were found for PC1/PC3 angle in the epicardium and for PC1/PC2 angle in the endocardium in SSS and for PC1/PC3 in the endocardium in SS. *p < 0.001.

**Table 1:** ANOVAs on angle differences.

Angle type	R-squared	*p*-value	Sig
Epi PC1/PC2 SSS	−0.00118	0.58627	
Epi PC1/PC3 SSS	0.11561	2.00E-16	*
Endo PC1/PC2 SSS	0.11208	2.00E-16	*
Endo PC1/PC3 SSS	0.01174	0.00455	*
Epi PC1/PC2 SS	0.02314	0.00011	*
Epi PC1/PC3 SS	0.04309	2.00E-16	*
Endo PC1/PC2 SS	0.03594	2.00E-16	*
Endo PC1/PC3 SS	0.1444	2.00E-16	*

**Figure 6:**
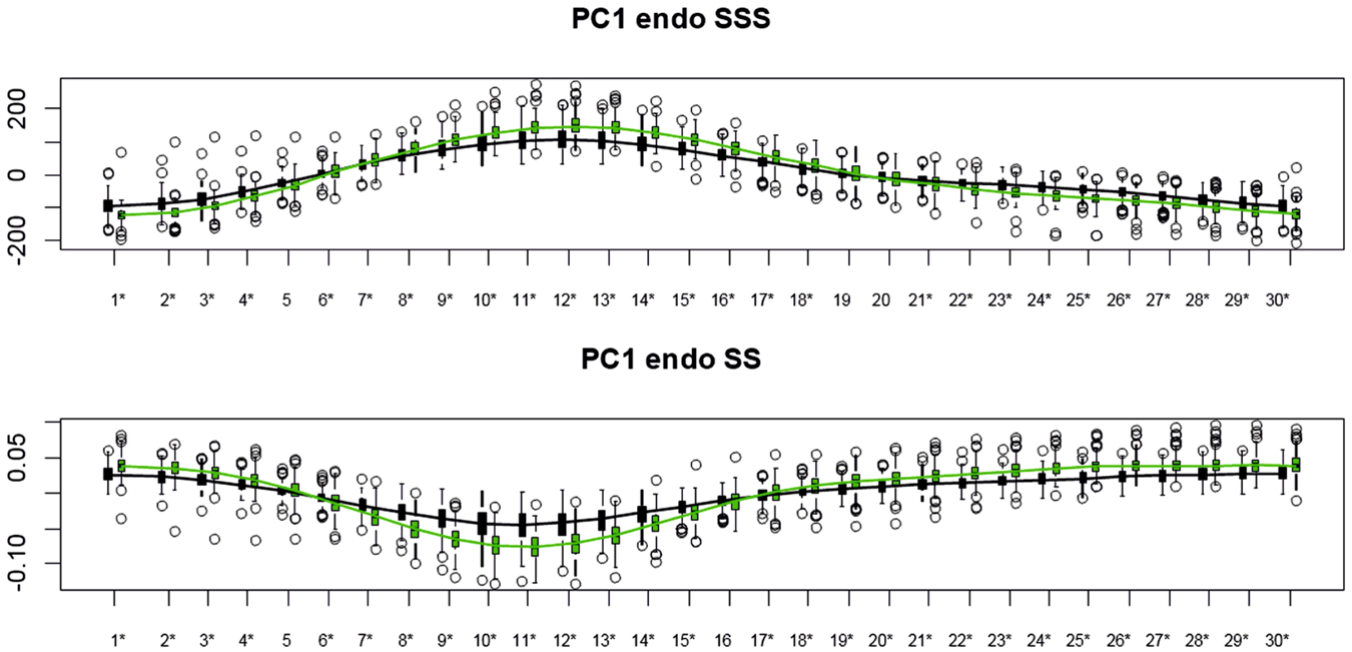
Temporal course of PC1 of the endocardial analysis in SSS (top) and SS (bottom). Asterisks indicate significance under ANOVA for the differences between Controls (green) and MI (black).

**Figure 7:**
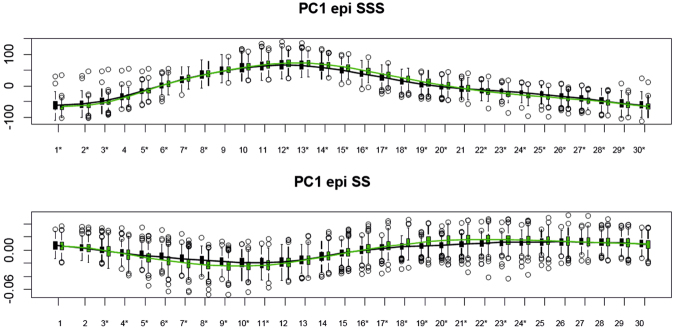
Temporal course of PC1 of the epicardial analysis in SSS (top) and SS (bottom). Asterisks indicate significance under ANOVA for the differences between Controls (green) and MI (black).

Figures 6 and 7 show the temporal trajectory of PC1, evaluated at all homologous times, and the distributions of Controls and MI as well as ANOVA significance results. The endocardium, in both systole and diastole, discriminated MI more than epicardium and SS performed better than SSS (see classification table). Table 2 shows the results relative to our subsplitting-based permuted SVM procedure. The best performance was reached by using the first 10 PC scores of LS + PCA in SS evaluated at all homologous times for the endocardium. SSS also performed very well for the endocardium, while the epicardium did not reach the same performance. The shape of the trajectory was a better indicator in SSS, for both epicardium and endocardium, while trajectory size was a better indicator in SS. Angles appeared to have similar performance in either SSS or SS.

**Table 2:** Mean classification performance of morphometric indicators over 1000 subsplitting-based permuted SVM simulations.

Independent variables	Accuracy	Specificity	Sensitivity	AUC
**Size and Shape Space**				
Endo angle PC1/PC2	0.63	0.49	0.78	0.68
Endo angle PC1/PC3	0.60	0.48	0.72	0.63
Endo trajectory size	0.75	0.69	0.81	0.78
Endo trajectory shape first 15 PCs	0.79	0.76	0.82	0.87
Linear Shift Endo PC1 evaluated at all homologous times	0.80	0.74	0.85	0.88
Linear Shift Endo PC2 evaluated at all homologous times	0.74	0.78	0.70	0.82
Linear Shift Endo PC3 evaluated at all homologous times	0.83	0.80	0.85	0.90
Linear Shift Endo PC1-PC10 evaluated at all homologous times	0.93	0.92	0.94	0.98
Epi angle PC1/PC2	0.51	0.48	0.53	0.52
Epi angle PC1/PC3	0.66	0.55	0.77	0.68
Epi trajectory size	0.58	0.60	0.56	0.60
Epi trajectory shape first 15 PCs	0.79	0.78	0.80	0.86
Linear Shift Epi PC1 evaluated at all homologous times	0.76	0.77	0.74	0.83
Linear Shift Epi PC2 evaluated at all homologous times	0.74	0.75	0.73	0.81
Linear Shift Epi PC3 evaluated at all homologous times	0.70	0.70	0.70	0.76
Linear Shift Epi PC1-PC10 evaluated at all homologous times	0.85	0.86	0.84	0.93
**Shape Space**				
Endo angle PC1/PC2	0.67	0.53	0.80	0.70
Endo angle PC1/PC3	0.68	0.54	0.83	0.71
Endo trajectory size	0.77	0.71	0.83	0.78
Endo trajectory shape first 15 PCs	0.72	0.74	0.71	0.79
Linear Shift Endo PC1 evaluated at all homologous times	0.83	0.79	0.87	0.92
Linear Shift Endo PC2 evaluated at all homologous times	0.79	0.81	0.76	0.87
Linear Shift Endo PC3 evaluated at all homologous times	0.81	0.84	0.78	0.89
Linear Shift Endo PC1-PC10 evaluated at all homologous times	0.94	0.93	0.96	0.98
Epi angle PC1/PC2	0.56	0.63	0.50	0.58
Epi angle PC1/PC3	0.59	0.64	0.55	0.62
Epi trajectory size	0.61	0.50	0.72	0.62
Epi trajectory shape first 15 PCs	0.75	0.75	0.75	0.84
Linear Shift Epi PC1 evaluated at all homologous times	0.76	0.79	0.73	0.84
Linear Shift Epi PC2 evaluated at all homologous times	0.73	0.78	0.67	0.79
Linear Shift Epi PC3 evaluated at all homologous times	0.67	0.70	0.65	0.73
Linear Shift Epi PC1-PC10 evaluated at all homologous times	0.86	0.88	0.83	0.93
**Traditional Indicators**				
Ejection fraction	0.85	0.83	0.88	0.90
ES volume	0.86	0.90	0.89	0.91
ED volume	0.80	0.78	0.84	0.84

## Discussion

The results presented in this work highlight the importance of evaluation of the whole LV deformational trajectory in MI. While classical shape analysis and classification procedures have been very effective in recognizing pathology using only ED and ES shapes, the pathophysiological interpretation of LV dynamics (whole motion) would be impossible by studying the ES and ED states only. In fact, when only ES and ED states were analyzed^6^ the training performance in total misclassification was lower (92%).

However, the sample and sample size were different and it would be difficult to compare these results. Another important result of this study is that the 4D trajectory analysis of MI patients showed impaired trajectory attributes in terms of shape, size and direction. This evidence is supported by similar analyses performed on hypertrophic cardiomyopathy (HCM)^2,3^ where it was found that HCM patients present very different ventricular and atrial trajectory attributes in comparison to controls. This leads to the conclusion that pathology does not affect only shape, as suggested previously^6,7^, but also the myocardial deformation that represents the morpho-functional expression of the cardiac cycle. As suggested in^6,7,10^, the deformation occurring between ED and ES is sufficient to classify MI with very high accuracy (>90%). These results are very good if compared with those coming from classification performed using the more classical indicators often used in the clinical literature to characterize MI, i.e. ED volume, ES volume and ejection fraction. We have shown that evaluating the entire trajectory in SS could improve this result. It is worth noticing that PC scores evaluated at all homologous times, even if they do not represent attributes of trajectories (shape of trajectory itself, orientation and size) can be genuinely considered “trajectory-based” as they acknowledge the entire course of cardiac cycle and, given that the grand mean was chosen as MR (as stated above), even the diastolic state is a “deformed” state. This is particularly evident in Figs 6 and 7 where the endocardium evaluated at ED, significantly discriminates, in both SSS and SS, Controls and MI. On the other hand, appreciating what occurs during the entire LV revolution could enable the use of motion attributes as additional physiological indicators, therefore improving the assessment of therapies and treatments. As suggested in^3^, these novel indicators of shape and trajectory could help quantify and guide therapeutic treatment after MI. Reverse remodeling is the aim of a pathology-specific therapy/treatment: by using these indicators, it becomes possible to have a population-based trajectory metric whereby MI treatment can be monitored as an impaired trajectory reverses back into a healthy one.

Moreover, the effect of alternative therapies can be quantified in terms of the strength of reverse remodeling.

Figure 1 shows that (1) Control cases have overall greater contractility, and (2) contraction is stronger around the papillary muscles and the apex in both groups (except for the septal area). Shape remodeling is inevitably coupled with motion remodeling thus constituting two different levels of morpho- functional abstractions. An interesting result arises from the comparison of trajectory size in epicardium and endocardium in both SSS and SS. MI possesses a much smaller endocardial trajectory, in comparison to Controls, than the epicardium. This is coherent with the notion that the endocardium captures most of size- shape changes occurring during contraction^2,14^. Again, morphometric analysis might enlarge the domain whereby information is obtained by 3D MR thus increasing the possibility of local analysis of the consequences of infarction, which might be developed in future studies.

In the SSS analysis, PC1 (Fig. 2) was related to size whereas PC2 appeared to relate to sphericity, in agreement with^10^. Thus, re-labelling SSS PC1 as “size” and SSS PC2 as “sphericity” in Fig. 2, the mean trajectories for both surfaces in Controls and MI show greater variability in size than sphericity, a result that might be explained by ventricular filling. Supplementary file 3 shows that SSS PC3 was related to the reduction in diameter of ventricular base that becomes wider at positive values. It is worth noticing here that only PC1 coincided in its positive/negative direction with the diastole/systole direction (=contraction); PC2 extremes (being orthogonal) corresponded to mid-systole and mid-diastole. The same holds for PC3. The endocardial trajectory size (Fig. 4) was overall larger than the epicardial one, a result in line with previous literature^15^. Also, the endocardium presented greater separation between Controls/MI than the epicardium. This  was expected in so far that most of the motion abnormalities in MI are typically more accentuated in the endocardium^16^ reinforcing the argument that at least when imaging by ultrasound, whereby virtually most of the epicardium is disregarded, most of the abnormalities can still be captured.

In terms of angles (Fig. 5), we can conclude that the trajectory modes relating to size and sphericity are not very effective in distinguishing between Controls/MI for the epicardium (except for PC1/PC3 angle), but are better for the endocardium, suggesting again that the endocardium carries most of the pathological motion expression for MI, particularly in SS. Our classification exercise highlights that PC scores of endocardium in SS, all taken together, have a very high classification power with a total accuracy larger than that found in^6^ for similar data. SS performed better than SSS and this holds also for the epicardium. Further work is needed to explain the lower classification performance using the shape of the trajectory (in SSS or SS), in comparison to PC scores evaluated at all homologous times. This might be interpreted as the expression of a morpho-functional dynamic constraint: a beating heart cannot have a trajectory shape too far from an elliptical one. The same was found previously^1–3^ for HCM, where the differences in trajectory shape were larger but still confined in the context of a closed approximately elliptical shape.

We have shown that there are intrinsic differences in the trajectory of shapes between MI and asymptomatic cases, defining a motion-based pattern analysis that can augment traditional shape analysis in the pursuit of objective and quantifiable clinical indicators of disease, for monitoring and treatment. The concept of reverse remodeling using the indicators of trajectories described here might be extended in different areas of ischemic heart disease. It is worthwhile to assess whether, by a population-based trajectory metric, impending conditions of congestive heart failure might be disclosed earlier than the appearance of systolic or diastolic incompetence. It is tempting to speculate that effective therapeutic prevention might then be applied if the strength of reverse remodeling should be shown. Moreover, myocardial indicators derived from MRI features could provide in the near future early clinical evidence of myocardial dysfunction. Characterization and quantification of different patterns found in shape and motion from readily available clinical diagnostic tools such as cardiac MR, enables efficient re-utilization of existing data and forms the basis of future automated detection. This will aid radiologists and cardiologists in achieving faster and more accurate diagnosis. While some simpler derived indices can have a clinical translation or connection (such as size and hypertrophy), more powerful (classification-wise) and statistically sound (non-correlated and homogenized) indices are hard to interpret clinically as they are data-driven. This is in essence the burden of translation studies in computational anatomy. We believe that the myocardial indices presented in this paper are powerful while still relating to interpretable motion characteristics.

## Methods

### Study population and geometric data

**Figure 8:**
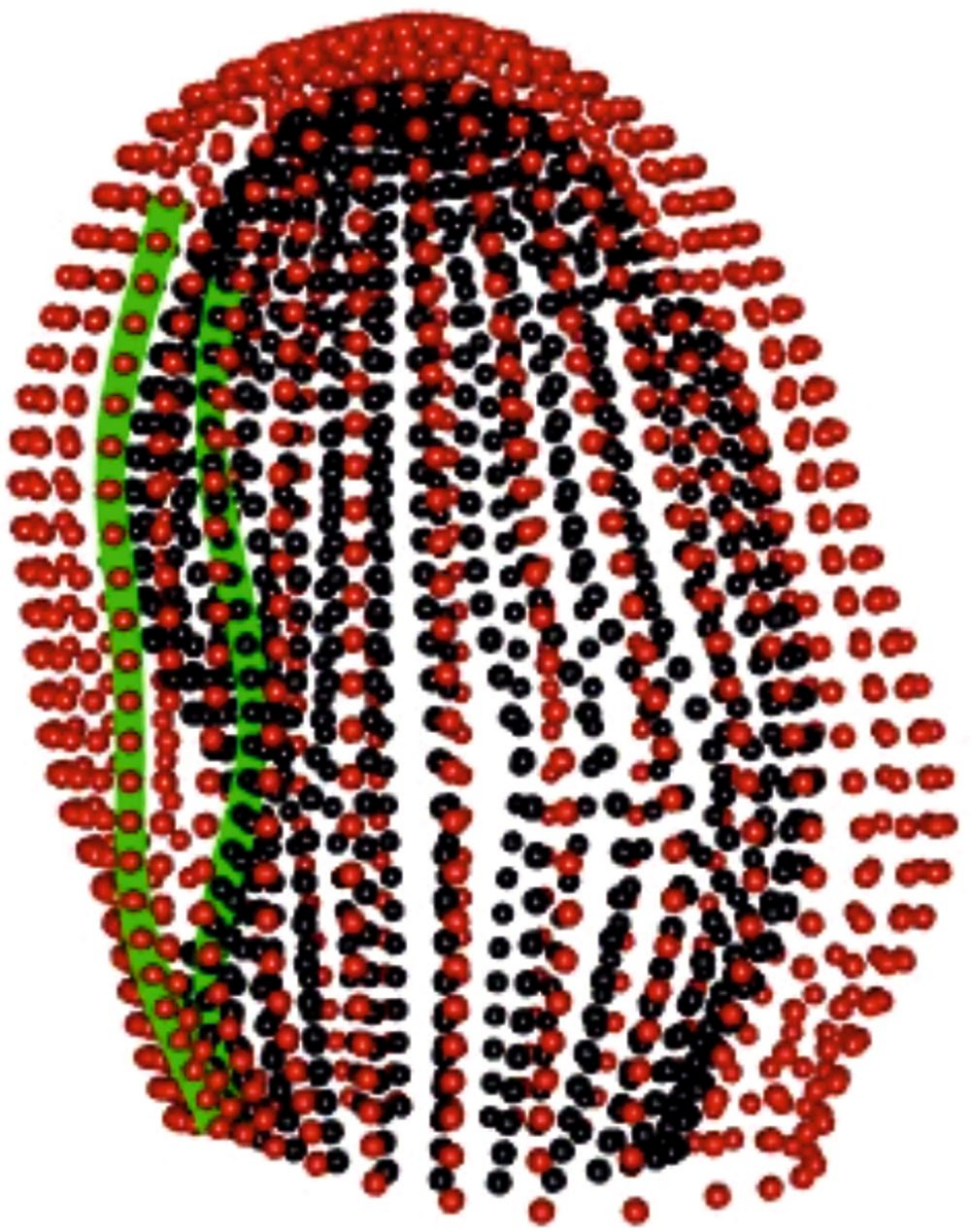
A configuration of one LV shape at end-diastole (a randomly chosen Control case). Red points (grey in printed version) are the epicardium, black points are the endocardium. Green lines (light grey in printed version) on the two layers indicate the middle of inter-ventricular septum. LV base is on the bottom, apex on top. Each of the 600 cases (300 MI and 300 Controls) comprises 30 shapes representative of one cardiac cycle for a total of 18,000 shapes.

This study was approved by the local institutional review boards (Johns Hopkins University School of Medicine NA_00031350; Northwestern University CR1_STU00000078; New Zealand Multi-region Ethics Committee MEC/08/04/052) and all participants gave written informed consent. All acquisitions were performed in accordance with relevant guidelines and regulations of the above mentioned Institutions. Our study comprised 300 asymptomatic volunteers and 300 patients with myocardial infarction randomly selected from the Cardiac Atlas Project (CAP) database^17^. The imaging protocol included cine images acquired in short-axis planes from the base of the heart to the apex and in three long-axis planes. All participants gave written consent compatible with data sharing. For each subject the CMR cine images were used to build finite element models as previously described^18^. Each shape was composed of 1,089 landmarks (assumed homologous^18^) for each of the epicardial and endocardial surfaces. Figure 8 shows an example shape of a control case from the database. Table 3 reports descriptive statistics for the population under study.

**Table 3:** Descriptive statistics. Continuous variables are expressed as mean ± standard deviation. Categorical variables are expressed as count. Actual sample size (thus excluding missing cases) is indicated for each entry.

Population features	MI	Controls
Total individuals acquired via MR	300	300
Gender	M = 238; F = 60; n = 298	M = 161; F = 139; n = 300
Age (years)	62.8 ± 10.7; n = 300	61 ± 9.7; n = 300
Systolic blood pressure (mmHg)	127.5 ± 20.13; n = 224	125.6 ± 23.6; n = 300
Diastolic blood pressure (mmHg)	73.9 ± 11.3; n = 223	71.5 ± 10.3; n = 300
Height (cm)	173.8 ± 9.8; n = 300	166 ± 9.7; n = 300
Weight (kg)	90.1 ± 19.1; n = 300	77.5 ± 16.0; n = 300
Heart rate (beats/s)	67.2 ± 12.3; n = 251	62.5 ± 9.9; n = 297
ED Volume (ml)	196.3 ± 52.9; n = 300	126.24 ± 26.3; n = 244
ES Volume (ml)	118.6 ± 48.9; n = 300	48 ± 16; n = 244
Ejection fraction (%)	41.3 ± 10.9; n = 300	62.4 ± 7.2; n = 244
Body mass index (kg/m^2^)	29.7 ± 5.6; n = 300	28.0 ± 5; n = 244
Body surface area (Du Bois formula)	2.0 ± 0.2; n = 300	1.84 ± 0.2; n = 244

### Temporal Registration

**Figure 9:**
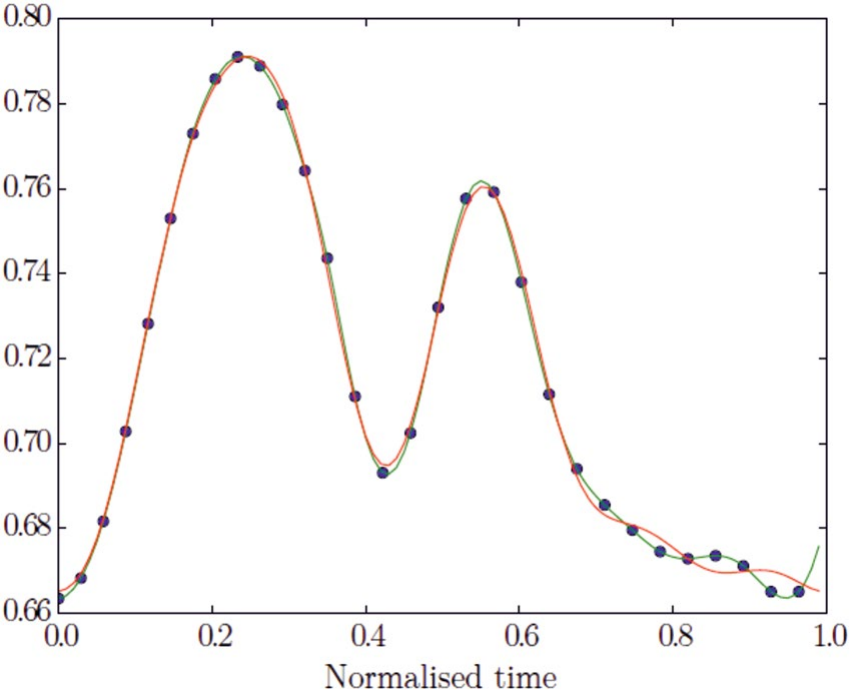
Example of a finite element parameter in time. The blue dots (black in printed version) represent the time- registered points at each frame, the green line (where dots are placed) the cubic B-spline, and the red line (where dots are not placed) the Fourier partial sums with 5 harmonics.

Each case was temporally registered following^19,20^ to obtain the same number of frames within a heart cycle standardized between 0% and 100% over the entire cardiac cycle. Finite element model shapes were thus temporally registered to obtain the same number of frames at the same temporal positions with respect to a normalized cardiac cycle. Briefly, a piece-wise time warp was applied to the discrete samples for each parameter and each patient, split by the end-systolic point which was aligned to be at 35% of the cycle. End- diastole was aligned to be continuous at 0 or 100% of the cycle. This was followed by a Fourier interpolation with five harmonics, providing a continuous and cyclic representation of each parameter in time. To compute the harmonic coefficients, a supporting B-spline function was used (Fig. 9). The resulting dataset was a series of 30 shapes (t = 1….30) for each patient each with end- diastole (ED) at t = 1 and end-systole (ES) approximately at frame 11 (Fig. 9). Note that the end-systolic time-frame was manually chosen by an expert analyst by observing the motion through the cardiac cycle, and did not coincide with the frame of minimum volume by numerical integration (since minimum volume was subject to noise in the surfaces during isovolumetric relaxation). Therefore after registration, end systole, defined now as the time at which volume is minimal, occurred among the 600 cases with the following variability of indices: t = 9: n = 4; t = 10: n = 14; t = 11: n = 82; t = 12: n = 207; t = 13: n = 197; t = 14: n = 77; t = 15: n = 13; t = 16: n = 3; t = 17: n = 3. Resulting data dimension was therefore n = 18,000 (=600 * 30), dim = 3, landmarks = 1,089 * 2. Further details and formulation can be found in^19,20^. We applied phenotypic trajectory analysis^1–4^ to these data.

### Geometric Morphometrics

When dealing with landmark-based data, Geometric Morphometrics (GM) is a powerful tool to interpret underlying processes of shape change phenomena^21–23^. GM eliminates non-shape variations, i.e. translation, rotation and optionally size via Generalized Procrustes Analysis^24^ (GPA). In the present study, all analyses were performed in both the Size and Shape Space (SSS) and in the Shape Space (SS). The two manifolds, in fact, illustrate different aspects of the relationship between shape and size, enabling a full evaluation of their ability to recognize pathology when evaluated under the GM paradigm. Usually, after GPA, Principal Component Analysis (PCA) is performed to find axes of maximal variation. However, in presence of multiple deformation series, such as individual heart cycles, GPA + PCA mix inter- and intra- individual variation thus impeding recognition of pure deformation patterns. If one is interested in identifying the pure deformation trajectory, a specific geometric tool, such as Parallel Transport (PT), is necessary to filter out inter-individual variation of shape.

### Parallel Transport via Linear Shift

**Figure 10:**
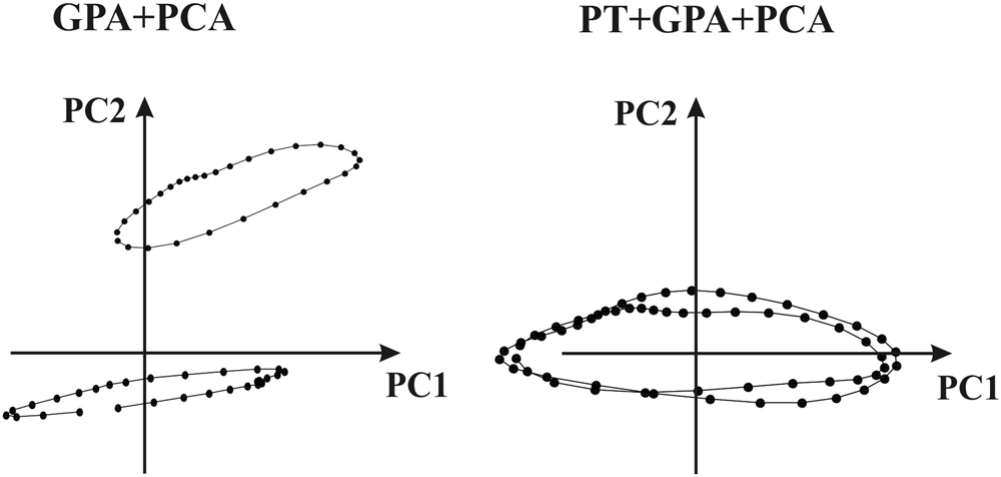
The meaning of PT when applied to two different trajectories. Left: When classic GPA + PCA are applied PC axes explain concomitantly inter- and intra- individual variation. Right: PT applied to the same data with deformation transported toward the Grand Mean; in this case PC axes explains solely the deformation (thus without initial inter- individual shape differences) applied to the consensus.

The motivation for applying PT is given in^25^. PT is a geometrical tool that allows transporting vectors from the tangent space at a point of a manifold to the tangent space at a different point. The manifold can be represented by the shape space or the size-and-shape space. When shapes are identified by homologous landmarks, the Levi Civita connection is often used to identify the geodesic between two shapes, and to transport deformations^26^. In GM^22,27^ the deformation between two different shapes is gauged using thin- plate splines (TPS), a function that minimizes the bending energy^21^. When configurations are centered, optimally rotated (minimizing Procrustes distances of the entire sample from the consensus) and optionally scaled to unit size, via GPA, shapes live on a curved manifold, i.e. SS or SSS if scaling is not performed. Shapes are then orthogonally projected on the tangent plane to the Grand Mean (often called “consensus”) in order to apply common multivariate methods. For small morphological variation (as in most biological investigations^28^) the Riemannian Procrustes distances are well approximated by the linear Euclidean distances. In the same way, the connection between two shapes can still be projected on the Euclidean tangent space, as an approximation of the actual geodesic. In this simplified case, the PT is computed by the deformation vector field between two optimally aligned shapes. Eventually, this vector field can be used to deform a third shape, i.e. a “mannequin” (called “common template”, CT, elsewhere), also optimally aligned with the source of the previous pair. The computation of the deformation vector field could be highly sophisticated if performed in the Riemannian manifold and review of the many proposed metrics is out of scope in this work. These deformations can be applied to the CT that is common to the entire sample. When using an Euclidean approximation, the deformation vector field can be estimated using Ordinary Procrustes Analysis (OPA) by aligning a target shape to a source (with or without scaling at unit size, see below). The deformation vector field results from the differences of the two shapes aligned via OPA. This approach is an Euclidean type of PT, based on simple operations as addition and subtraction, and has been effectively applied the past^1–4,6^ under the name “Linear Shift” (LS). Basically, it estimates deformations within the cycle of each subject between observed shapes and a properly chosen individual-specific local template (LT). LTs can be the local means, i.e. the means of each individual, and they can be found via separate GPAs for any subject. To estimate deformations, each shape belonging to each individual is aligned via OPA with its proper LT. Deformations are then computed via subtraction. Then all deformations are “transported” toward the CT via addition. It is worth noticing that LTs were preliminary aligned with CT (via OPA) before deformations estimation and their successive transport. After that a common GPA is performed. In this way starting inter-individual shape differences are filtered out and the pure deformations can be studied. This procedure is relatively easier to implement and it does not require the computation of complicated Riemannian connections^29,30^. It enables the comparison of deformation paths occurring within each group, filtering out any inter-group differences. Specifically, we identify as CT the Grand Mean of the entire sample, and use LS to apply individual deformation, occurring within each cycle, to the CT. We find the CT (=Grand Mean) via a common GPA (in either SSS or SS) performed on the entire sample. This GPA only serves to find the CT. All LTs are aligned with it, via OPA, and then LS is performed. When using the Grand Mean as a CT, instead of ES, ED and ES appear as deformed states. Therefore the composition of deformation analysis consists of: (GPA in SSS or SS to find the Grand Mean = CT) + (separate GPAs in SSS or SS for each individual to find LTs) + (separate OPAs to align each LT with CT) + (LS in SSS or SS) + (GPA in SSS or SS) + PCA. The outcome of this procedure is a series of deformations of the CT that properly manages rotations. Close points in the resulting PCA plot do not correspond to individuals that have the same shape but indicate individuals that are experiencing the same deformation. Further details about LS can be found in^1,2,6,31^. Figure 10 shows, synthetically, the meaning of PT when applied to two trajectories in the size and shape space.

### Trajectory analysis

**Figure 11:**
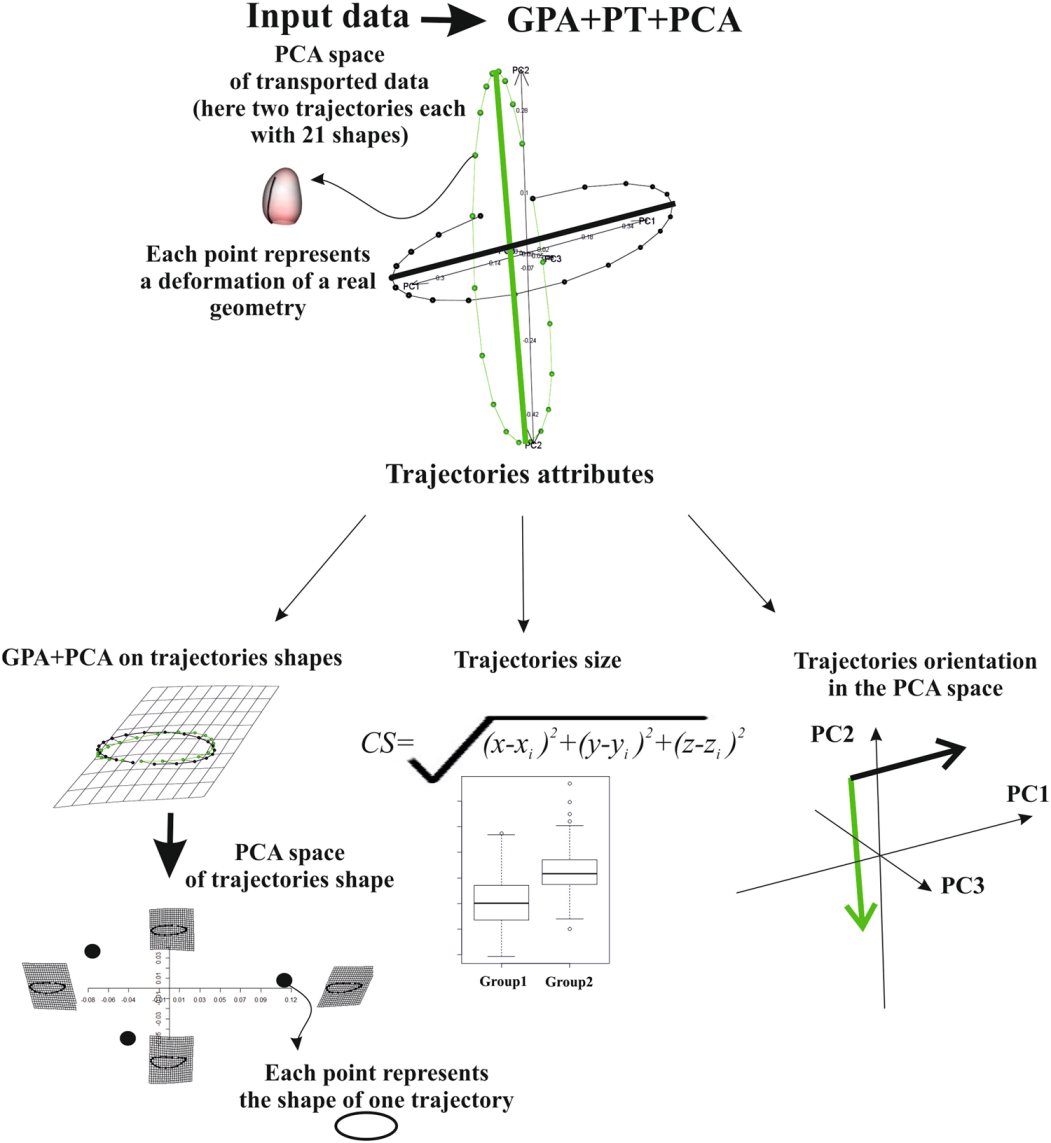
Flowchart for the trajectory analysis using a platonic example.

In the present contribution, we aim to explore the deformational attributes of LV motion for MI and Control groups. This means that the LV trajectory must be assessed. Figure 11 shows that a morphological trajectory can be evaluated by means of at least three attributes: size, orientation and shape. These attributes can be estimated by treating the trajectory itself as a geometric shape. The “landmarks” of this shape can be the scores in the space identified by the first three principal component scores^1–4,6^ arising from the application of (LS) + (GPA) + PCA to the data interpolated as described above. In fact, previously, shapes were interpolated at individual-specific electro-mechanical times^1–4,6^.

When using MR cardiac data, this is not possible hence the temporal registration procedure explained above was used in order to obtain the same number of shapes. Shape scores in PCA were treated as homologous landmarks in trajectory shape analysis. MANOVA was used to assess differences between groups (Control vs MI) in the shape of trajectory. We additionally tested (for endocardium and epicardium) the multivariate dispersion differences in trajectory shape between Control and MI as in^1^. We used the first 15 PC scores that we anticipate  they explain about 98% of total variance for both endocardium and epicardium. Analyzing the shape scores in this manner allows for the characterization of the normal and abnormal domains in shape space.

ANOVA was performed on the centroid size’s trajectories to assess  trajectory' size differences. The angle of trajectories was estimated by calculating the angles in PC1/PC2 and PC1/PC3 spaces for vectors connecting principal component scores at ED and ES for each individual. These angles indicate the morphological direction connecting the end-diastolic and end-systolic states. An impaired condition affecting ES state inevitably places pathological subjects in different regions of the morphospace thus generating significantly different angles. ANOVAs were then performed on these angles. Attributes of trajectories arising from both SSS and SS manifolds were compared and tested for their classification performance. The same holds for PC scores evaluated for all individuals at each homologous time. It is important to note that the shape of the trajectory was reconstructed using the first 3 PCs after the LS procedure. These collectively explain a certain amount of total variance. However, the procedure we describe here can be effectively applied to more than 3 PCs. GPA on trajectory shapes can be performed on hyper-shapes identified in n dimensions (using n PC components). In this case the hyper-shape cannot be simply illustrated and the interpretation of results requires a higher level of mathematical abstraction. This could limit the accessibility of this approach to a wider scientific community. Further studies could investigate whether hyper-shapes (n > 3) could improve the present analysis.

### Classification

Several myocardial motion indicators were extracted from cardiac MR shape data. Using the procedures described previously to register shapes in the temporal, spatial and morphological domains, we propose a classification procedure aimed at recognizing pathology. In^6,10^ it was shown that Support Vector Machine (SVM) applied to LS + PCA data outperformed other classification methods (such as discriminant analysis, logistic regression and random forest among others). This result was obtained using data arising from the same databases used in the present paper but only from systolic and diastolic states without considering the entire cardiac trajectory. Aiming to exploit the full potential of a trajectory-based approach, we adopt here the same SVM strategy^6^ for testing classification performance. The 300 Controls and 300 MI cases were randomly split into a training dataset of 200 Control and 200 MI to be used for learning and a test dataset of 100 Control and 100 MI used for classification. This was done 1,000 times in order to produce a large amount of classification exercises performed on unknown data. At each run, the 100 Control and 100 MI randomly chosen test cases were classified using the function estimated using the corresponding training dataset and total accuracy, specificity, sensitivity and Area Under the Curve (AUC) of the Receiver Operating Characteristic (ROC) curve were recorded. The mean values over all runs were retained as representative of global classification performance. To compare these novel morphological indicators against traditional indicators of cardiac function, we performed the same classification procedure using ED and ES volumes, and ejection fraction (EF).

### Data availability statement

Data can be obtained through the Cardiac Atlas Project (www.cardiacatlas.org).

## Electronic supplementary material

Supplementary file 1

Supplementary file 2

Supplementary file 3

Supplementary file 4

Supplementary file 5

Supplementary file 6
